# Comparative epidemiology of Middle East respiratory syndrome coronavirus (MERS-CoV) in Saudi Arabia and South Korea

**DOI:** 10.1038/emi.2017.40

**Published:** 2017-06-07

**Authors:** Xin Chen, Abrar Ahmad Chughtai, Amalie Dyda, Chandini Raina MacIntyre

**Affiliations:** 1 School of Public Health and Community Medicine, University of New South Wales, Sydney, NSW 2052, Australia; 2 College of Public Service and Community Solutions, Arizona State University, Tempe, AZ 85287, USA

**Keywords:** Middle East respiratory syndrome coronavirus, epidemiology, emerging infectious disease

## Abstract

MERS-CoV infection emerged in the Kingdom of Saudi Arabia (KSA) in 2012 and has spread to 26 countries. However, 80% of all cases have occurred in KSA. The largest outbreak outside KSA occurred in South Korea (SK) in 2015. In this report, we describe an epidemiological comparison of the two outbreaks. Data from 1299 cases in KSA (2012–2015) and 186 cases in SK (2015) were collected from publicly available resources, including FluTrackers, the World Health Organization (WHO) outbreak news and the Saudi MOH (MOH). Descriptive analysis, *t*-tests, Chi-square tests and binary logistic regression were conducted to compare demographic and other characteristics (comorbidity, contact history) of cases by nationality. Epidemic curves of the outbreaks were generated. The mean age of cases was 51 years in KSA and 54 years in SK. Older males (⩾70 years) were more likely to be infected or to die from MERS-CoV infection, and males exhibited increased rates of comorbidity in both countries. The epidemic pattern in KSA was more complex, with animal-to-human, human-to-human, nosocomial and unknown exposure, whereas the outbreak in SK was more clearly nosocomial. Of the 1186 MERS cases in KSA with reported risk factors, 158 (13.3%) cases were hospital associated compared with 175 (94.1%) in SK, and an increased proportion of cases with unknown exposure risk was found in KSA (710, 59.9%). In a globally connected world, travel is a risk factor for emerging infections, and health systems in all countries should implement better triage systems for potential imported cases of MERS-CoV to prevent large epidemics.

## INTRODUCTION

MERS-CoV first emerged in the Kingdom of Saudi Arabia (KSA) in 2012^1^ and has since spread to 26 countries.^2^ By far, the greatest burden of disease is located in KSA, and most cases in other countries have not resulted in large satellite epidemics. The exact origin of MERS-CoV remains unknown, but the transmission pattern and evidence from virological studies suggest that dromedary camels are the major reservoir host,^3, 4, 5, 6^ from which human infections may sporadically occur through zoonotic transmission. Human-to-human transmission also occurs in healthcare facilities and communities.^7, 8, 9^ Globally, as of 16 January, 2017, a total of 1879 laboratory-confirmed cases of MERS-CoV and at least 659 deaths have been reported to the WHO.^10, 11^ The case fatality rate (CFR) in patients (35%) is higher than that of Severe Acute Respiratory Syndrome (SARS) (9.6%).^12, 13^ In contrast to MERS-CoV, the SARS epidemic, which exhibited an increased estimated reproductive number *R*_0_ of ~2, peaked, waned and ended within 8 months. MERS has a lower *R*_0_, estimated to be closer to 1. In addition, MERS has paradoxically persisted with a largely sporadic pattern for over four years.^14, 15^ Among the global MERS cases, males (63%)^16^ are more affected than females, with a male to female ratio of 1.7:1.^17^ The mean age of all cases reported worldwide is 49 years, and most cases are between 50 and 59 years of age.^17^

The largest MERS outbreak to date outside KSA occurred in SK,^18, 19^ with 186 cases and 39 deaths (CFR: 21%) reported from May to July in 2015. All cases (excluding the index case, who had traveled from the Middle East) were linked to a single-chain of transmission and were associated with healthcare facilities, owing to the lack of awareness of MERS-CoV at the index hospital and inappropriate triage and infection control.^20^ Males were more affected than females in SK, comprising 59% of total laboratory-confirmed cases^21^ and 66.7% case fatalities.^22^ Prevention measures, including placing over 3000 people in quarantine and closing 700 schools, were implemented for outbreak control.^23^ The aim of this study was to compare the epidemiology of MERS-CoV in KSA and SK before 2016.

## MATERIALS AND METHODS

A database of MERS cases was created by using data collected from the onset of the first case in June 2012 to December 2015 in KSA and SK. The confirmed cases of MERS-CoV in KSA and SK were sourced from FluTrackers. To validate the data, we assessed and enhanced the data set with more detailed information from reports published by the WHO, Promed Mail and local organizations, such as the Saudi MOH, during the outbreak. The data collected included case list number, demographic characteristics (e.g., age, sex and healthcare worker status), date of notification, comorbidity, date of symptoms onset, date of first hospitalization, date of laboratory confirmation, current status, date of outcome, contact history and nationality. We categorized cases into six age groups as follows: <30 years, 30–39 years, 40–49 years, 50–59 years, 60–69 years and ⩾70 years. The proportion of cases in each age group was calculated by dividing the number of cases in each age group by the number of total cases, then multiplying by 100. The sex-specific CFR per age group was calculated by dividing the number of deaths among males or females in an age group by the total number of cases limited to the one sex in that age group, then multiplying by 100. On the basis of different contact or exposure risk factors, the contact history was classified into camel-linked (contact with camels and camel products), sheep-linked (contact with sheep and sheep products), hospital-linked (contact with diseased patients or healthcare workers, or healthcare facilities which had MERS-CoV outbreak), community-linked (contact with diseased family members or friends) and unknown (no contact history or investigation ongoing).

Cases with missing values for age, sex, healthcare worker, comorbidity, date of symptoms onset and contact history were excluded from the analysis. Data were missing for 113 cases for all variables,^24^ 36 cases for age, 49 cases for sex, 19 cases for fatality, 20 cases for healthcare worker status, 121 cases for comorbidity and six cases for contact history in KSA. In addition, data were missing for 16 cases regarding healthcare worker status and 145 cases regarding comorbidity in SK. The data were used to plot epidemic curves. Descriptive analysis was conducted to calculate the mean value of continuous variables (e.g., age). *t*-tests, Chi-square tests and binary logistic regression were used to compare the mean values of age and the proportions of categorical variables (e.g., sex, healthcare worker, comorbidity, contact history) on the basis of nationality. The analysis was conducted in SPSS version 22. A *P*-value<0.05 was considered statistically significant.

## RESULTS

**Figure 1:**
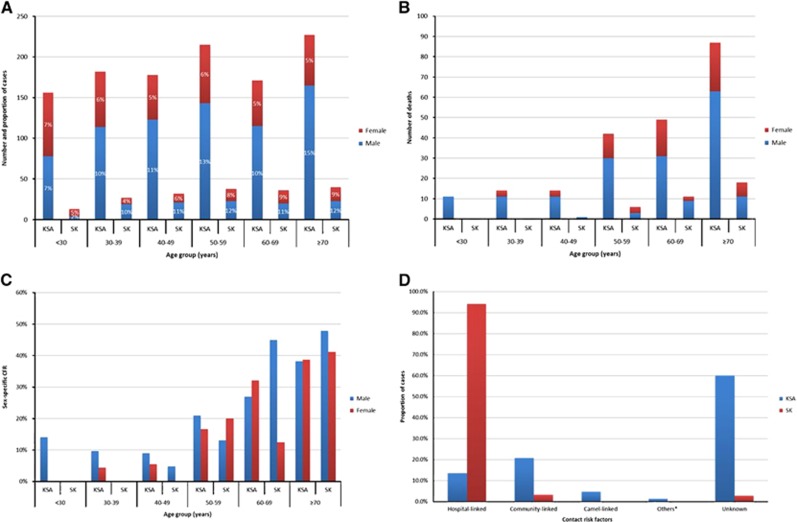
The distribution of MERS cases and deaths in KSA (2012-2015) and SK (2015)^#^. (**A**) Number and proportion of MERS cases by sex and age group; (**B**) Number of deaths by sex and age group; (**C**) Sex-specific CFR by age group; (**D**) MERS cases by contact risk factors. *Others include contact risk factors of sheep-linked, camel and sheep-linked, camel and community-linked and camel, sheep and hospital-linked.

A total of 1299 cases (1186 cases in analysis) from KSA and 186 cases from SK were reported from 2012 to 2015. On the basis of the available data, the comparison of demographics and other characteristics between KSA and SK is presented in Table 1. The mean age of MERS cases was 51 years in KSA, and 54 years in SK. In both countries, most cases were reported in the age group ⩾70 years (KSA: 227 of 1186 cases, 19.1% and SK: 40 of 186 cases, 21.5%) followed by the age group 50–59 years (KSA: 215 of 1186 cases, 18.1% and SK: 38 of 186 cases, 20.4%) (Figure 1A). Overall, there was a higher frequency of males among cases in KSA (741 of 1137, 65.2%) than in SK (110 of 186, 59.1%); however, no significant differences in sex distribution were found between the two countries. The overall CFR was 19.2% (228 fatalities of 1186 cases) in KSA and 19.4% (36 fatalities of 186 cases) in SK. The age-specific CFR increased by age in both counties, and the highest values were reported in the age group ⩾70 years (Figures 1B and 1C). In terms of sex-specific CFR, there was no significant difference between KSA and SK. No significant difference was found in the proportions of infected healthcare workers between KSA (157 of 1166, 13.5%) and SK (26 of 170, 15.3%). Rates of MERS cases with comorbidity were also higher in males in both KSA (460 of 657, 70.0%) and SK (21 of 35, 60.0%). No significant differences in sex-specific comorbidity rates were observed between KSA and SK.

MERS cases in KSA had various contact risk factors before symptom onset (Figure 1D), including contact with camels and camel products (e.g., camel raw milk) (*n*=59, 5.0%), sheep and sheep products (*n*=5, 0.4%), nosocomial MERS cases or hospitals having MERS outbreak (*n*=158, 13.3%), and diseased family members or friends (*n*=245, 20.7%), and a majority had unknown risk factors (*n*=710, 59.9%). In addition, several cases had more than one risk factor, including contact with both camels and sheep (*n*=6, 0.5%), camels and confirmed human cases (*n*=2, 0.2%), and the mixture of several contact types (*n*=1, 0.1%). In SK, most cases had a contact history in hospital facilities (*n*=175, 94.1%), whereas only six cases (3.2%) were infected with MERS-CoV through community contacts. The differences in the rates of community-linked, hospital-linked or unknown risk factors between KSA and SK were statistically significant (*P*<0.001).

**Figure 2:**
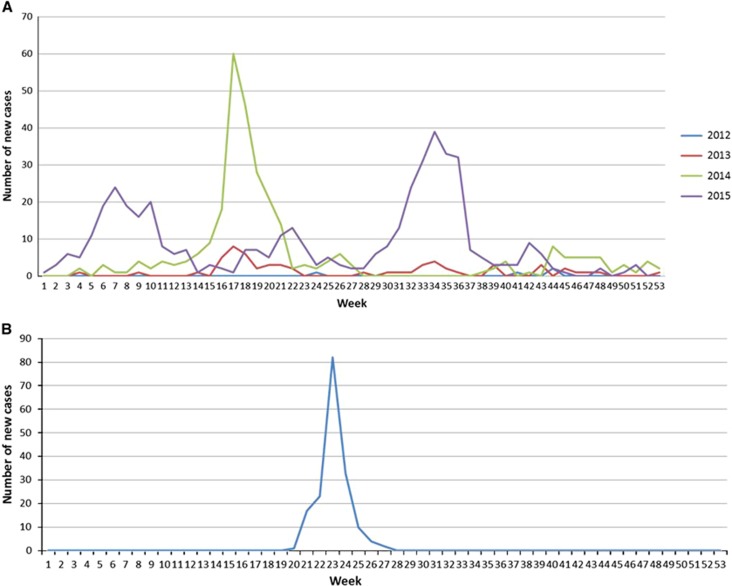
Number of MERS cases by the week of onset of symptoms. (**A**) Kingdom of Saudi Arabia, 2012–2015 (*n*=805); (**B**) South Korea, 2015 (*n*=172).

On the basis of the reported date of onset of symptoms, the epidemic curve in KSA from 2012 to 2015 by week is shown in Figure 2A. During the four years after the emergence of MERS-CoV, several peaks were observed in week 18 in 2014 and weeks 7, 10, 34 in 2015. In addition, numerous clusters and sporadic cases were noted with no clear seasonality, comprising a very protracted and mixed pattern. The epidemic curve of MERS-CoV in SK in 2015 (Figure 2B) exhibited a classic epidemic curve over a short time (8 weeks) with a small number of early cases beginning in week 20 followed by a rapid rise in new cases over two weeks, an epidemic peak at week 23 and then a decline in cases ending at week 28.

## DISCUSSION

We compared the characteristics of 1299 laboratory-confirmed MERS cases in KSA with 186 cases in SK from 2012 to 2015, by using publicly available data. The main differences between the two countries included the slightly older age of cases in SK, the epidemic pattern and the risk factors for infection. SK had a predominantly nosocomial transmission pattern, with >90% of cases acquiring infection in the health system. In contrast, in KSA, the occurrence of MERS-CoV was characterized by a very mixed epidemic pattern^15^ with a diversity of risk factors for disease, and >60% of cases had no known risk factor for infection. This finding is consistent with other published studies indicating a high proportion of cases without animal or nosocomial contact.^8, 15, 25, 26^ Interestingly, despite the large difference in nosocomial cases between SK and KSA, the rate of healthcare worker cases was not significantly different between countries, thus highlighting the high-occupational health risk that MERS-CoV poses to health workers. The epidemic pattern in KSA has been substantially different each year from 2012 to 2015 and exhibited an absence of seasonality, varying timing of annual peaks and a mixture of sporadic and epidemic patterns. The large epidemic peaks in KSA were not well explained by the estimated *R*_0_ of 0.6–1.3.^27, 28, 29^ In contrast, the epidemic curve in SK was clearly epidemic in pattern.

The demographic features of cases (such as age and sex) were similar and have been described previously in KSA^28, 29, 30^ and SK;^20, 21, 31, 32^ however, cases were slightly older in SK. In addition to the higher proportion of male cases with underlying comorbidities, the reasons for males being more at risk may be related to various socio-cultural behaviors. One possible explanation for the excess of male cases is that females are more likely to adopt hygienic measures and health-seeking behaviors, as observed during past influenza pandemics, such as that of H1N1.^33^ Women are also more likely in KSA to wear face veils, thus potentially decreasing exposure. However, this cultural practice is not the case in SK, which also had a male predominance.^34^ The predominance of male cases may also be associated with the high prevalence of smoking, particularly in middle-aged males in the Republic of Korea,^32^ which has been considered an independent risk factor of respiratory infections, such as pneumonia.^35^ However, the association between smoking and MERS-CoV infection requires more supportive evidence.

Sporadic and clusters of MERS cases had been reported from KSA, which were probably due to contact with camels and other animals. However, >60% of cases in KSA had no clear history of exposure to camels or other animals, and a study of camel handlers revealed no evidence of MERS-CoV infection despite handling of camels with MERS-CoV infection.^36^ Despite the epidemic arising in SK from a single traveler,^18^ there have been no epidemics to date in KSA or other countries arising from mass gatherings, such as the annual Hajj pilgrimage (week 43 in 2012, week 41 in 2013, week 40 in 2014 and week 39 in 2015), a phenomenon that is difficult to explain.^37^ In addition, previous evidence has demonstrated multiple genetic strains in a single nosocomial outbreak at Al Ahsa Hospital in KSA,^38^ a finding inconsistent with a short-time frame single epidemic. The identification of multiple genetic variants over a longer period of time may be expected but is unexpected in a single outbreak occurring within a matter of days or weeks.

All cases in SK (excluding the index case) were associated with a single chain of transmission and healthcare facilities.^18, 39^ The index case was a 68-year-old man who traveled to the United Arab Emirates (29–30 April), Saudi Arabia (1–2 May), Qatar (2–3 May), and Bahrain (4 May) and returned to Seoul on the same day without a history of exposure to camels or contact with MERS cases.^39^ His respiratory illness developed on 11 May. He received treatment in Hospital A (local clinic) on 12, 14 and 15 May; Hospital B (hospitalized) from 15 to 17 May; and Hospital C and Hospital D (hospitalized) from 17 May. He was diagnosed with MERS-CoV on 20 May.^20^ A range of hospitalized patients, healthcare workers and family members were exposed during this period, thus leading to the nosocomial disease outbreak in SK. The MERS-CoV isolated from inpatients formed a single monophyletic clade with high similarity to strains from Riyadh.^40^ An unreported sequence was detected in the MERS-CoV circulating in SK,^41^ thus suggesting increased genetic variability and mutation rates during the outbreak in 2015.

This study is limited by missing data and the use of only publicly available data. Of the 1299 cases in KSA, ~10% (113 of 1299) of cases were excluded, owing to missing data for all variables; thus, our database represented 90% of reported laboratory-confirmed cases in KSA. Despite our best efforts to obtain information through various public resources, the data still contained gaps for some variables in KSA (age: 36 of 1186, 3% sex: 49 of 1186, 4% fatality: 19 of 1186, 2% healthcare worker: 20 of 1186, 2% comorbidity: 121 of 1186, 10%) and SK (healthcare worker: 16 of 186, 9% comorbidity: 145 of 186, 78%). In this study, we classified the contact history into five types, including camel-linked, sheep-linked, hospital-linked, community-linked and unknown. The unknown was defined as the MERS cases with no contact history or cases in which the investigation was ongoing. The corresponding data were taken from official websites, including those of the WHO and the Saudi MOH, as well as local news and medical reports. However, we were unable to identify specified risk factors or contact history for these 710 cases of unknown type. In addition, there appeared to be some inconsistencies in reporting that affected this study, because publicly available data were used. The fatalities calculated in this analysis totaled 228 cases with a CFR of 19%, a value different from that reported by the Saudi MOH (43%).^42^ This difference may be due to the incomplete information of deaths provided by Saudi MOH at the time. If more complete data become available, further analysis on mortality-related risk factors is recommended. This study highlights the value of publicly available data to improve understanding of complex emerging infections. We were able to consolidate and enhance available data through searching multiple data sources, but there is a global need for making high-quality disease surveillance data publicly available on a routine basis for transparency and enhanced disease control efforts.

The transmission in SK was found to be predominantly hospital linked and a classic epidemic over a short period of time, thus highlighting the importance of hospital triage protocols for treating emerging infectious diseases. In the case of SK, low awareness of MERS-CoV at the index hospital and poor triage and infection control resulted in a large outbreak, which might have otherwise been prevented. We ranked the risk of travel-acquired MERS-CoV infections by country, on the basis of airline travel patterns and frequencies, which can be used to prioritize hospital screening protocols for countries at highest risk.^26^ SK was in the top 50 countries at risk in this analysis and ranked at number 38. In contrast, India ranked first but has not experienced an imported case of MERS-CoV. This information again highlights the need for a global approach to mitigate the spread of emerging infectious diseases and the risk that all countries face in an interconnected world of travel-related epidemics.

In conclusion, on the basis of the comparative epidemiology of MERS-CoV in KSA and SK, both countries had differing risk factors and epidemic patterns, thus adding to the complexity of this disease. The varying and complex epidemiology in KSA is consistent with multiple introductions, which may comprise a mix of animal-to-human, human-to-human (healthcare-acquired, community-acquired), and other modes of transmission. A large proportion of KSA cases have unknown exposure, thus warranting further study. MERS-CoV has an epidemic pattern that has varied from country to country and has disproportionately affected KSA for reasons that are not yet fully understood. Further research, enabled by high quality surveillance data, is required to understand and mitigate the risk factors for MERS-CoV.

## Figures and Tables

**Table 1:** Demographics and other characteristics of laboratory-confirmed MERS cases in the Kingdom of Saudi Arabia (2012–2015) and South Korea (2015)

**Variables**	**Kingdom of Saudi Arabia (*n*=1186)^a^**	**South Korea (*n*=186)**	***P*-value**
Mean age (range)^b^	51 (0–109) years	54 (16–87) years	0.023
			
*Sex*^c^			
Male	741/1137 (65.2%)	110/186 (59.1%)	0.111
Female	396/1137 (34.8%)	76/186 (40.9%)	

*Sex-specific fatalities*^d^			
Male	157/741 (21.2%)	24/110 (21.8%)	0.484
Female	60/396 (15.2%)	12/76 (15.8%)	0.550
Healthcare worker^e^	157/1166 (13.5%)	26/170 (15.3%)	

*Comorbidity*^f^			
Male	460/657 (70.0%)	21/35 (60.0%)	0.211
Female	197/657 (30.0%)	14/35 (40.0%)	

*Contact History*			
Camel-linked	59/1186 (5.0%)	0/186 (0%)	NA
Sheep-linked	5/1186 (0.4%)	0/186 (0%)	NA
Hospital-linked	158/1186 (13.3%)	175/186 (94.1%)	<0.001
Community-linked	245/1186 (20.7%)	6/186 (3.2%)	<0.001
Camel and sheep-linked	6/1186 (0.5%)	0/186 (0%)	NA
Camel and community-linked	2/1186 (0.2%)	0/186 (0%)	NA
Camel, sheep and hospital-linked	1/1186 (0.1%)	0/186 (0%)	NA
Unknown	710/1186 (59.9%)	5/186 (2.7%)	<0.001

Abbreviation: not applicable, NA.

a Missing data for 113 cases in KSA.

b Age: 36 cases in KSA were unavailable.

c Sex: 49 cases in KSA were unavailable.

d Overall fatalities: 19 cases in KSA were unavailable.

e Healthcare Worker: 20 cases in KSA and 16 cases in SK were unavailable.

f Comorbidity: 121 cases in KSA and 145 cases in SK were unavailable.
